# Optical projection tomography permits efficient assessment of infarct volume in the murine heart postmyocardial infarction

**DOI:** 10.1152/ajpheart.00233.2015

**Published:** 2015-06-12

**Authors:** X. Zhao, J. Wu, C. D. Gray, K. McGregor, A. G. Rossi, H. Morrison, M. A. Jansen, G. A. Gray

**Affiliations:** ^1^BHF/University Centre for Cardiovascular Science, Queen's Medical Research Institute, University of Edinburgh, Edinburgh, United Kingdom;; ^2^Edinburgh Preclinical Imaging, BHF/University Centre for Cardiovascular Science, Queen's Medical Research Institute, University of Edinburgh, Edinburgh, United Kingdom;; ^3^Clinical Research Imaging Centre, University of Edinburgh, Edinburgh, United Kingdom;; ^4^Centre for Inflammation Research, University of Edinburgh, College of Medicine & Veterinary Medicine, Queens Medical Research Institute, Edinburgh, United Kingdom; and; ^5^MRC Human Genetics Unit, MRC Institute of Genetics and Molecular Medicine, Western General Hospital, University of Edinburgh, Edinburgh, United Kingdom

**Keywords:** mouse myocardial infarction, late gadolinium enhancement magnetic resonance imaging, troponin I, Masson's trichrome, ejection fraction

## Abstract

The extent of infarct injury is a key determinant of structural and functional remodeling following myocardial infarction (MI). Infarct volume in experimental models of MI can be determined accurately by in vivo magnetic resonance imaging (MRI), but this is costly and not widely available. Experimental studies therefore commonly assess injury by histological analysis of sections sampled from the infarcted heart, an approach that is labor intensive, can be subjective, and does not fully assess the extent of injury. The present study aimed to assess the suitability of optical projection tomography (OPT) for identification of injured myocardium and for accurate and efficient assessment of infarct volume. Intact, perfusion-fixed, optically cleared hearts, collected from mice 7 days after induction of MI by coronary artery occlusion, were scanned by a tomograph for autofluorescence emission after UV excitation, generating >400 transaxial sections for reconstruction. Differential autofluorescence permitted discrimination between viable and injured myocardium and highlighted the heterogeneity within the infarct zone. Two-dimensional infarct areas derived from OPT imaging and Masson's trichrome staining of slices from the same heart were highly correlated (r^2^ = 0.99, *P* < 0.0001). Infarct volume derived from reconstructed OPT sections correlated with volume derived from in vivo late gadolinium enhancement MRI (r^2^ = 0.7608, *P* < 0.005). Tissue processing for OPT did not compromise subsequent immunohistochemical detection of endothelial cell and inflammatory cell markers. OPT is thus a nondestructive, efficient, and accurate approach for routine in vitro assessment of murine myocardial infarct volume.

## Abstract (precis)

*Optical projection tomography permits rapid high-resolution imaging of intact murine heart in vitro and identification of tissue heterogeneity within individual optical slices of postmyocardial infarction hearts. Infarct volume derived from >400 slices correlates with in vivo magnetic resonance imaging and avoids the need for histological staining of multiple physical sections*.

## NEW & NOTEWORTHY

*Optical projection tomography permits rapid high-resolution imaging of intact murine heart in vitro and identification of tissue heterogeneity within individual optical slices of postmyocardial infarction hearts. Infarct volume derived from >400 slices correlates with in vivo magnetic resonance imaging and avoids the need for histological staining of multiple physical sections*.

murine models are widely used to study injury, repair, and remodeling of the heart following myocardial infarction (MI), as well as in the development of novel therapies to prevent progression to heart failure (9). The extent of infarct injury in the immediate postischemic period and after infarct healing is a critical determinant of long-term structural and functional outcome. Accurate determination of infarct size is thus vital in interpretation of these outcomes. The current gold standard for infarct measurement in clinical (5) and experimental studies (31) is magnetic resonance imaging (MRI) using a contrast agent such as gadolinium (Gd) to enhance the image of the infarcted area [late Gd enhancement (LGE)]. This permits three-dimensional (3D) assessment of infarct volume. However, as LGE-MRI requires expensive specialist equipment, assessment of experimental infarct injury more typically relies on 2D histological assessment in a single section or multiple sections collected from the infarct area. Histological methods are time consuming, fail to provide information on infarct volume, and are less sensitive than 3D assessment for detection of changes in the extent of injury during healing (3). The increased error arising from dependence on histological analysis in a limited range of sections can result in a need for increased groups sizes to adequately power studies for statistical analysis, and consequently higher animal use.

Optical projection tomography (OPT) was originally developed for enhanced imaging of whole mount mouse embryos and can be applied to image biological samples up to a depth of 15 mm with high resolution (22). Briefly, once optically cleared, the sample is suspended in an index-matching liquid to reduce light scattering. As the sample moves 360° around the scanner, 400 images are collected and virtual sections are then reconstructed to acquire a detailed 3D image (15, 21a). The samples are illuminated by visible light for transmission imaging to distinguish anatomical structures. Alternatively emission imaging can record light emitted following excitation at varied wavelengths, detecting either endogenous fluorescence or fluorescence emitted by fluorophores placed within the tissue e.g., associated with specific antibodies. Anatomical structure can be derived from both due to distinct differences in light transmission and autofluorescence between tissues. Since the launch of this technique, OPT has been applied in developmental biology (10, 13, 23), for gene-expression analysis (25), to localize labeled cells within a tissue (30), and to screen normal and abnormal anatomy (12, 24). Kirkby et al. (15) recently described an elegant application of OPT to provide 3D quantification of murine neointimal and atherosclerotic lesions.

The components and fluorescence characteristics of tissue in the healing MI are distinct from those of healthy myocardium. The present study therefore aimed to investigate the potential of OPT for identification and quantification of the extent of infarct injury during the infarct healing phase 7 days after induction of MI by left coronary artery ligation. The outcomes were compared with those from in vivo LGE-MRI and 2D histological assessment in hearts from the same mice. As tissue processing is required to obtain OPT images, hearts collected following OPT were also sectioned to test their suitability for subsequent immunohistochemical studies.

## MATERIALS AND METHODS

### 

#### Animals.

**Fig. 1.**
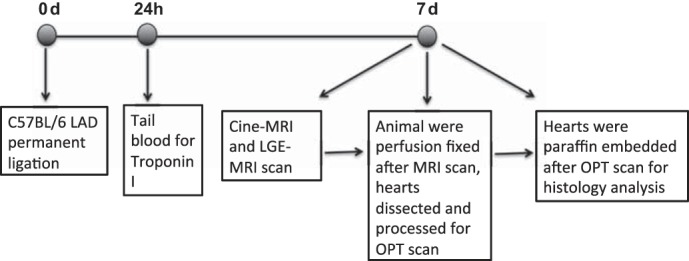
Flowchart of experimental protocol. LGE, late gadolinium enhancement; OPT, optical projection tomography.

We obtained 10 male C57BL/6 mice, age 12–14 wk, from Harlan UK. All animal experiments were performed in accordance with the Animals (Scientific Procedures) Act (UK), 1986 and approved by the University of Edinburgh Preclinical Ethical Review Committee. All mice underwent the protocol described in Fig. 1.

#### Induction of MI.

After administration of analgesic (buprenorphine, 1 mg/kg sc), each mouse's chest was anesthetized with isoflurane (2%), ventilated (stroke volume 240 μl, 120 breaths/min), and opened at the left third intercostal space to expose the heart. The pericardium was partially removed, and a 6-0 prolene suture was placed under the left anterior coronary artery and tied. The distance between the ligature position and the apex was varied to achieve a variety of infarct sizes for analysis. Successful ligation was indicated by blanch of the ventricle. After closing the chest, we injected 1.0–1.5 ml prewarmed 0.9% saline sc into the left flank to aid recovery, and mice were allowed to recover overnight on a heated pad.

#### Cardiac troponin I.

Twenty-four hours after induction of ischemia, 30 μl blood was collected by tail nick into a tube containing 30 μl 3.2% sodium citrate for determination of cardiac troponin I (cTnI) by high-sensitivity ELISA (Life Diagnostics), as an index of the extent of myocardial injury following coronary artery ligation. The limit of detection for cTnI was 0.156 ng/ml. One mouse had a plasma level of cTnI <5 ng/ml and was excluded from the study as a missed ligation.

#### MRI.

Mice were scanned 7 days after induction of MI in a 7T MRI system (Agilent Technologies) with a quadrature birdcage coil (33 mm inner diameter). We instrumented isoflurane-anesthetized mice to assess respiration rate and ECG for signal gating; body temperature was maintained at 37°C by a hot air blower with feedback control.

For LGE-MRI, 0.5 mmol/kg gadoterate meglumine (Gd-DOTA; Dotarem, Guerbet, France) was administered intraperitoneally. Scout images were acquired to guarantee correct positioning of consecutive scans, and flip angle calibration was performed 35–40 min after Gd-DOTA injection; cardiac-gated short-axis inversion recovery gradient echo images covering the entire left ventricle (8–10 slices) were acquired with the following parameters: repetition time ∼410 ms (depending on heart rate); echo time 1.94 ms; inversion time 350 ms; acquisition matrix 128 × 128; field of view 30 mm × 30 mm; slice thickness 1 mm; pulse angle 90°; number of signal averages 4. This time point was chosen to obtain the best contrast between remote and infarcted myocardium as described (3).

Following LGE, MR cine-images were acquired for functional analysis using a cardiac- and respiratory-gated gradient echo pulse sequence with the following parameters: repetition time 6 ms; echo time 1.8 ms; acquisition matrix 192 × 192; field of view 30 mm × 30 mm; number of time frames 16; slice thickness 1 mm; pulse angle 15°; number of signal averages 2. Images were acquired in the 2-chamber (1-slice), 4-chamber (1-slice), and short-axis (8–10 slices) orientations. The entire MRI session lasted ∼60 min. Ejection fraction, end-diastolic volume, end-systolic volume, and stroke volume were assessed, in a blinded manner using *CAAS* software (Pie Medical, Maastricht, the Netherlands). The papillary muscles and pericardium where present after surgery were excluded from analysis for all data sets.

#### OPT imaging.

Following MRI, hearts were fixed in situ by perfusing anesthetized mice via the abdominal aorta with 10 IU/ml heparin in 0.9% saline followed by 10% phosphate buffered formalin. The hearts were postfixed in 10% phosphate buffered formalin overnight then rehydrated in PBS. Hearts were embedded in 1.5% low-melting-point agarose (Invitrogen, UK), dehydrated in 100% methanol (twice, for 24 h each), and optically cleared by immersion in a mixture of 1 part benzyl alcohol (Sigma-Aldrich, UK) to 2 parts benzyl benzoate (BABB, Acros Organics, UK) for 48 h. Cleared hearts were scanned in a calibrated Bioptonics 3001 tomograph (Bioptonics, UK). Optical magnification was determined to provide the smallest voxel size while allowing the entire heart to be visualized, which resulted in a voxel of 17.05^3^ μm^3^. All hearts were imaged through emission imaging after UV illumination (470 nm excitation filter with 40 nm band pass; emission filter: 515 nm long pass; 1,024 × 1,024 pixel resolution) for ∼10–15 min per heart to generate 400 projections per scan with a 0.9° rotation.

Tomographic reconstruction by Hamming-filtered back-projection was performed using NRecon software (Skyscan, Belgium). The quality of reconstruction and 3D views were manually verified using DataViewer software (Skyscan).

#### Preparation of histological sections after OPT scanning.

After OPT scanning, hearts were returned to 100% methanol to remove benzyl alcohol benzyl benzoate (twice, for 24 h each) before rehydration. Hearts were then cut on their short axis at the level of the ligature and embedded in paraffin. Hearts were sliced transversely from the ligation to the apex with a microtome at 5 μm thickness with an interval of 300 μm between each sections, as described (26). We acquired six to eight sections from the ligation to the apex and selected four for comparison with OPT optical sections. Sections were mounted on glass slides and underwent Masson's trichrome staining for infarct identification. One heart was damaged during sectioning and was removed from the study.

#### 3D infarct volume measurement by MRI and OPT.

**Fig. 2.**
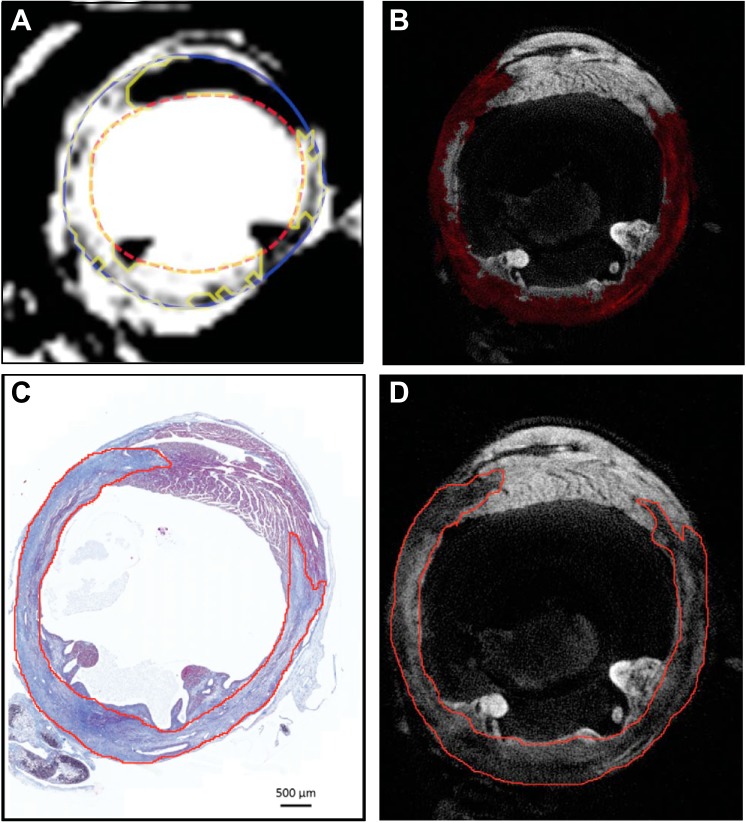
3D (*A, B*) and 2D (*C, D*) quantification of infarct size acquired by MRI, OPT, and histology. This figure shows a section through the same heart viewed by MRI (*A*), OPT (*B* and *D*), and histology (*C*). For 3D infarct volume measurement, in images generated by T2* weighted LGE-MRI (*A*), semiautomatic thresholding permitted separation of viable myocardium (black) from infarcted myocardium [white after injection of gadolinium (Gd) contrast, with yellow contour]. In OPT reconstructions, infarct threshold setting was based on weaker emission from the infarct scar, highlighted in red (*B*). For 2D infarct area measurement, the infarct area is identified by blue staining in Masson's trichrome-stained sections (*C*). *D*: the same section viewed using OPT, in both the infarct is traced in red. In all images, signal associated with the pericardium and the fluid within the pericardial sac (in in vivo MR only) is manually excluded for analysis.

For 3D quantification and comparison of infarct size, LGE-MRI image files were first converted to Digital Imaging and Communications in Medicine (DICOM) format, the international standard for medical images and related information. For LGE-MRI, infarct was identified by semiautomatic threshold setting with *CAAS* software (Pie Medical) to allow quantification of viable and infarcted myocardium (Fig. 2*A*). For OPT imaging, 3D volume quantification was performed using Analyze 11 (AnalyzeDirect, Overland Park, KS). In brief, reconstructed tomographic image files were imported into Analyze, then intensity thresholds were adjusted manually for one slice to achieve the optimum delineation between viable and infarcted tissue. A standard region growing algorithm within Analyze then allowed identification of regions of interest (ROIs) for infarct volume within that slice. Areas away from the infarcted ventricle that had similar pixel intensity e.g., the pericardium, were manually excluded (see Fig. 2*B*). This process was repeated at intervals of 10–20 slices throughout the infarct (260–340 slices), and then ROIs were propagated automatically for all slices in between the manually adjusted slices, forming an infarct ROI for the whole left ventricle (LV). Similarly, LV volume was determined by manually segmenting epicardial and endocardial contours at intervals of every 50 slices (360–450 slices), with automatic propagation of contours for all slices in between. LV volume was then calculated as the difference between epicardial and endocardial volumes. The pixel intensity threshold of the infarcted area was adjusted in both software packages so that only the lesion was selected. Infarct size was calculated as a proportion of LV volume (infarct volume/total LV volume × 100, LV volume includes viable myocardium above ligation). All OPT analyses were performed in a blinded manner and independently of MR images.

#### 2D infarct area quantification by histology and OPT.

Histological sections were scanned (Axio Scan. Z1, Carl Zeiss, Germany). Images were initially processed by Zen blue 2012 software (Carl Zeiss) and then saved as a compatible JPG format for subsequent analysis in ImageJ 1.47v for Mac OS X. Histological sections corresponding to OPT optical sections were identified based on the distance from the ligation, in the *z*-axis and on localization with respect to the papillary muscle. Area measurements in Image J were performed in a blinded manner. Infarct area in both histological sections and OPT tomographic reconstructions were traced manually (Fig. 2, *C* and *D*). We achieved LV area measurement by tracing the endocardial and epicardial borders. Infarct size was calculated as sum of infarct areas/sum of LV areas from four histological sections or OPT reconstructed optical sections and multiplied by 100. All histological analysis was performed in a blinded manner.

#### Post-OPT immunohistochemistry.

Immunohistochemistry was performed for Mac-2 (total macrophages) and CD31 (endothelial cells) to test the retention of immunoreactivity in sections following OPT processing and imaging. In brief, rehydrated paraffin sections were incubated with primary rat anti-mouse/human Mac-2 antibodies (1:4,000 dilution overnight; Cedarlane, Ontario, Canada) and rabbit anti-mouse CD31 antibodies (1:50 dilution overnight; Abcam, UK) before incubation with biotinylated rabbit anti-rat IgG (1:200 dilution 40 min; Vector Labs, UK) or biotinylated goat anti-rabbit IgG secondary antibodies (1:200 dilution 40 min; Vector Labs), respectively. The sections were then treated with avidin and biotinylated horseradish peroxidase macromolecular complex (3 drops/slide 30 min; Vector Labs) before coloring by 3,3′-diaminobenzidine substrate kit (100 μl/slide 3–5 min; Vector Labs).

#### Statistical analysis.

Statistical analysis was performed using Prism 6f for Mac OS X (GraphPad Software). 2D and 3D infarct size quantification by OPT, histology, and MRI were analyzed by correlation analysis (Pearson correlation coefficient, r^2^), followed by Bland-Altman analysis. *P* < 0.05 was considered statistically significant.

## RESULTS

### 

#### Coronary artery ligation induces a range of infarct injury.

All 10 animals undergoing coronary artery ligation surgery survived until the end of the study. The plasma concentration of cTnI varied between 33.54 and 91.29 ng/ml in blood collected 24 h after induction of MI demonstrating a range in the extent of cardiac injury attained in response to coronary artery ligation. Troponin I was <5 ng/ml in one mouse, which was subsequently found to be a sham with no discernable infarct; the heart from this mouse was removed from subsequent analysis.

#### Fluorescent emission imaging with excitation at 470 nm is optimal for identification of myocardial injury.

**Fig. 3.**
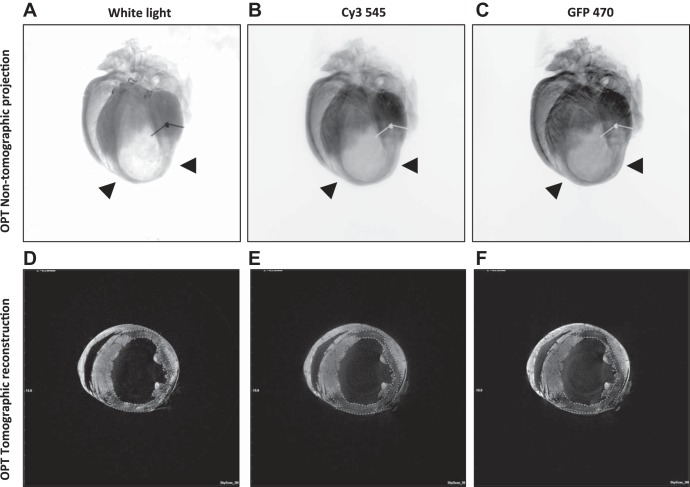
Optimization of image acquisition for identification and measurement of infarct regions in the mouse heart postmyocardial infarction (MI). In nontomographic projection images of an infarcted mouse heart, thinning of infarcted area is clearly visible (black arrowheads) when images were obtained using whether white (visible, *A*) or fluorescent light (*B*, 545 nm; *C*, 470 nm), compared with the darker viable myocardium. In tomographic reconstructions (*D–F*), the infarct region (dark, dashed contours) is optimally separated from the brighter viable myocardium, when applying the 470 nm excitation filter (*F*).

To determine the suitability of OPT for the identification of infarcted myocardium, 7 day postinfarct hearts were scanned for visible light transmittance and at two different fluorescent emission wavelengths (Fig. 3). While scanning in the visible (white) light channel, the contrast between viable and infarcted myocardium was sufficient to allow image reconstruction. However, emission from collagen or other fibrotic elements was too bright and widely distributed to permit threshold setting and analysis (Fig. 3, *A* and *D*). In both fluorescence emission channels, the viable myocardium was observed to have strong autofluorescence and was clearly delineated from infarct regions. Better resolution in the noninfarcted tissue and optimal discrimination between infarct and healthy myocardium were achieved following excitation at 470 nm (Fig. 3, *C* and *F*), and fluorescent emission imaging with excitation at 470 nm was thus adopted for all further investigations.

#### OPT reconstruction and histological assessment provide comparable measures of infarct area in 2D sections.

**Fig. 4.**
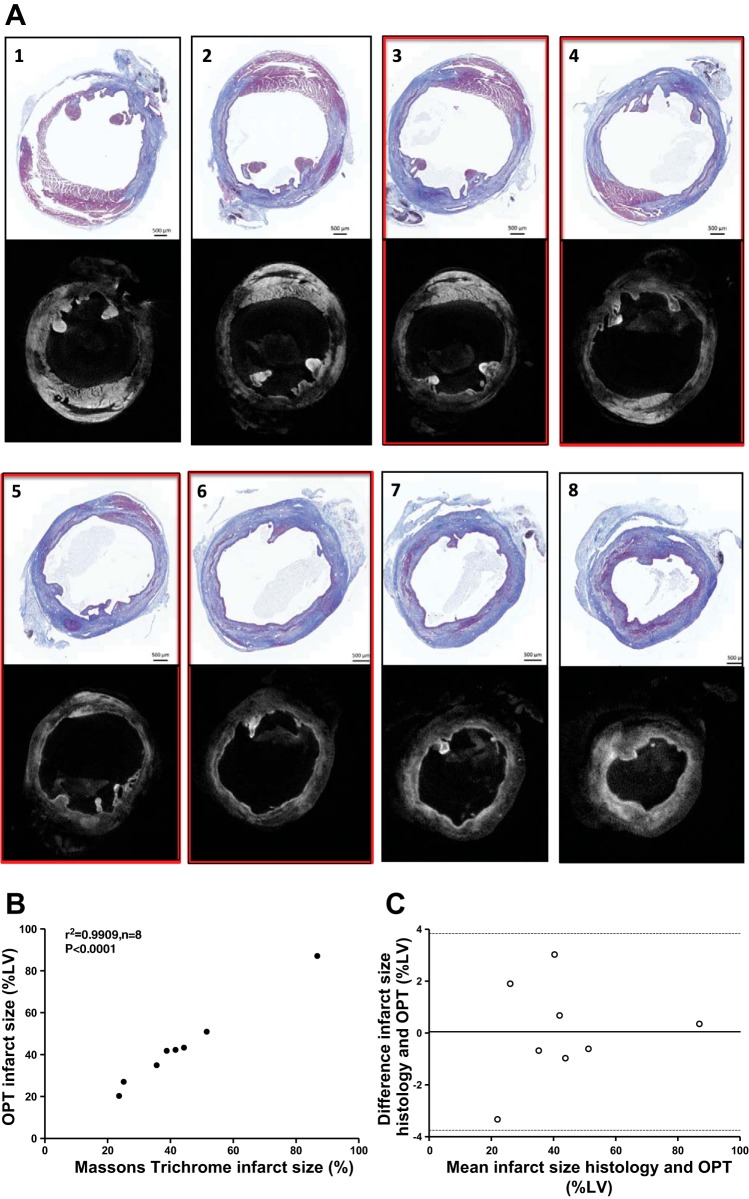
Comparison of infarct size determined by OPT and histology in 2D sections. Short-axis sections from the same heart (*A*) are shown from the level of ligation (*1*) to the heart apex (*8*), viewed both as OPT tomographic reconstructions (*bottom* panels) and corresponding Masson's trichrome-stained histological sections (*top* panels). Viable myocardium is bright (OPT) or red/pink (histology), while infarcted tissue is dark (OPT) or blue (histology). Correlation (*B*) and Bland-Altman analysis (*C*) were performed to compare the 2 methods on infarct size measurement. *Sections 3–6* were used for 2D determination of infarct size (highlighted by red rectangles).

2D OPT reconstruction provided remarkably similar identification of infarct injury to Masson's trichrome-stained histological sections subsequently collected at corresponding levels of the same heart (Fig. 4*A*). A wide range of infarct sizes were acquired (Fig. 4*B*), and significant correlation was evident between infarct sizes derived from the average of four histological sections sampled from the infarct and four optical OPT slices (r^2^ = 0.99, *P* < 0.0001, *n* = 8). Bland-Altman analysis indicated a bias of only 0.046% LV, and all plots were within the ±1.96 SD limits of agreement (−3.75 to 3.84% LV) (Fig. 4*C*).

#### Infarct characteristics and infarct volume data generated by OPT correlate with LGE-MRI.

**Fig. 5.**
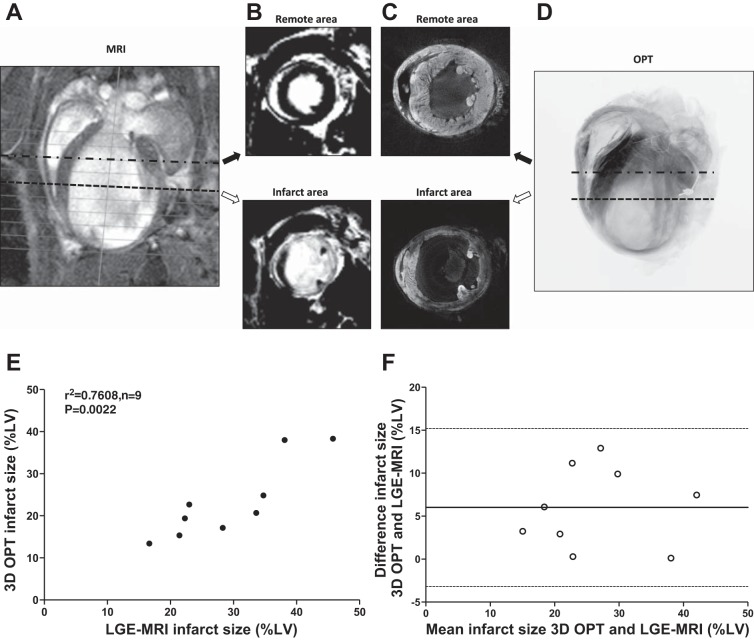
Comparison of LGE-MRI and 3D OPT for infarct detection and volume assessment. A longitudinal 4-chamber image obtained by cine-MRI (*A*) was used to position collection of short-axis sections imaged by LGE-MRI (*B*, white region indicates infarction while viable myocardium in black) or OPT reconstructions (*C*, infarct dark and viable myocardium bright). *D*: the OPT nontomographic projection of the same heart. Correlation (*E*) and Bland-Altman analysis (*F*) were performed to compare the 2 methods (*n* = 9 mice).

Noninvasive cardiac LGE-MRI was performed in mice 7 days after induction of MI and immediately prior to collection of hearts for OPT imaging. Four-chamber images were generated from both techniques (Fig. 5, *A* and *D*), and 2D sections collected from both indicated matching localization and extent of injury (Fig. 5, *B* and *C*). The average infarct volume was 29.3 ± 3.1% LV when assessed by LGE-MRI and 23.3 ± 3.0% LV when assessed by OPT (*P* = 0.93, *n* = 9). Correlation analysis shows that the two methods correlated strongly for infarct volume measurement (R^2^ = 0.76, *P* = 0.002, *n* = 9, Fig. 5*E*). Bland-Altman analysis indicates no obvious systematic bias in measurements of infarct volume (6.01% LV, *n* = 9), and all plots were within the limits of agreement (± 1.96 SD, −3.19 to 15.21% LV, Fig. 5*F*).

#### Infarct volume measured by 3D OPT or LGE-MRI correlates significantly with left ventricular ejection fraction.

**Fig. 6.**
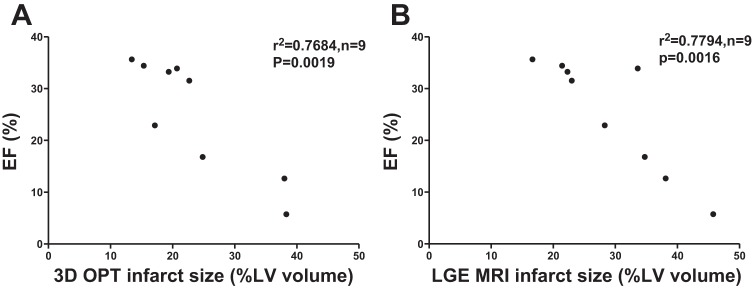
Correlation of systolic function with infarct volume assessed by 3D OPT and LGE-MRI. Left ventricular ejection fraction (LVEF) was calculated as an index of systolic function by cine-MRI. Infarct volume generated from 3D OPT (*A*) and LGE-MRI (*B*) are both highly correlated with LVEF, *n* = 9.

Left ventricular ejection fraction (LVEF) was measured by cine-MRI at the time of LGE-MRI assessment as an index of systolic function. Infarct volume derived from OPT was found to correlate highly with function (R^2^ = 0.7684, *P* = 0.0019, Fig. 6*A*) and to a similar extent as correlation of infarct volume generated from LGE-MRI and LVEF (R^2^ = 0.7794, *P* = 0.0016, Fig. 6*B*).

#### Tissue processing for OPT does not compromise later immunohistochemical staining of the healing MI.

**Fig. 7.**
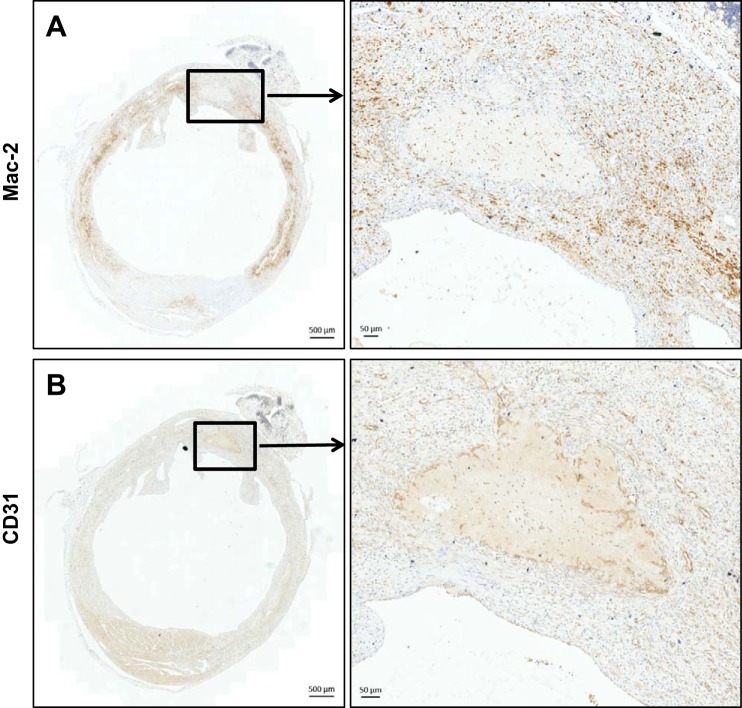
Immunohistochemical staining of heart sections are not compromised by prior tissue preparation for OPT scanning. Sections, collected from paraffin-embedded hearts of 7 days postinfarct mice, were immunostained for detection of macrophages (*A*, Mac-2) and endothelial cells (*B*, CD31). Scale bars are 500 μm in *left* panels and 50 μm in *right* panels.

In hearts previously subjected to OPT scanning, immunohistochemical staining using antibodies directed against markers of macrophages (Mac-2) and endothelial cells (CD31) occurred with the expected intensity and distribution (Fig. 7).

## DISCUSSION

This study demonstrates the suitability of OPT as a novel method for visualization of myocardial injury and for quantification of infarct volume in the healing murine heart following MI. Detection of differential tissue fluorescence in multiple “optical” slices allows discrimination between infarcted and viable myocardium in the intact heart, permitting 3D reconstruction and calculation of infarct volume without the need for physical sectioning (see video in the supplementary file).^1^ The resulting data correlate well with output from in vivo assessment by MRI, and the tissue remains suitable for subsequent immunohistochemistry or histology where required.

Infarct size is a key determinant of structural and functional outcome following MI. In the clinical setting, acute injury in patients is assessed by plasma biomarkers, such as troponin (18), and, where it is available, by MRI or PET, allowing calculation of infarct volume (27, 32). Advanced in vivo imaging can also be applied in preclinical studies (6, 17), but the equipment required is expensive and not widely available. Most preclinical MI studies depend on histological assessment of injury in tissue sections samples from the infarct, either acutely, with triphenyl tetrazolium chloride [TTC, (20)] or after the infarct scar has developed, with Masson's trichrome staining (26). Unless multiple sections are collected throughout the length of the infarct, these histological approaches give at best an estimate of infarct injury rather than an actual measurement of infarct volume.

In the present study the inherent autofluorescent characteristics of the myocardium have been exploited to permit identification of healthy myocardium and to distinguish it from damaged infarct tissue that has distinct emission characteristics. The OPT scanning technique permits collection of multiple “optical slices” in different planes. Analysis of individual optical sections collected in the short axis reveals a close correlation between areas of infarct identified by fluorescence emission after excitation at 470 nm with those identified subsequently in sections from the same hearts by histological staining with Masson's trichrome. The precise physical principles on which autofluorescence at 470 nm depend are not clear. Contractile filament proteins and components of cellular metabolism e.g., reduced forms of NADPH and FAD, are endogenous fluorophores; the latter may contribute to the signal arising from cells that had been metabolically active (21). Emission spectra can also be altered by relative content of collagen and elastin, also endogenous fluorophores that are likely to be more prevalent in the infarct tissue visualized here by OPT (21). The capacity to detect healthy tissue is particularly important in the murine infarcts where the infarct area is often thinned and delineation of structures within the thinned area can be challenging. In the present study hearts were collected at 7 days after induction of MI, when the healing infarct contains both viable and granulation tissue, in order to test the potential for OPT to discriminate between them. One of the key benefits of OPT is the ability to identify this heterogeneity and then to use information from hundreds of slices to reconstruct a complete 3D image of the heart identifying the full extent of the infarct and allowing calculation of volume (see video in supplementary file). Generating comparable data from histological sections would be extremely labor intensive and time consuming. In comparison, tissue preparation for OPT is straightforward, and imaging acquisition times are short, allowing high throughput of hearts. While not included in the present study, it will be of interest in future to assess the potential for OPT to discriminate between viable and nonviable myocardium in the early stages after infarction, compared with the established TTC method (21), as well as in more advanced repair when the infarct area is thinned and replaced largely by scar.

The gold standard for collection of 3D infarct volume is considered to be MRI, particularly when combined with LGE (14). In the present study LGE-MRI was used to detect infarct volume in vivo immediately prior to collection of the heart for in vitro OPT. Analysis revealed a significant correlation between volumes calculated by the two different modalities, although the actual volumes calculated by OPT tended to be lower than those from MRI. There are several potential reasons for this discrepancy. It has been suggested that LGE-MRI overestimates infarct injury because of the kinetics of Gd distribution and accumulation in areas of edema (7, 19). This error may be magnified by dependence of the MRI volume calculation on data from nine short-axis slices, rather than the hundreds of slices collected by OPT. The limited resolution of MRI relative to OPT also makes it less likely to identify viable myocardial tissue within the infarct and as a result exclude it from the infarct area. However, like histology, OPT may also underestimate the extent of injury. Fixation and methanol dehydration during tissue preparation have been associated with tissue shrinkage (8). This could lead to underestimation of relative infarct volume, or area in histological analysis, particularly if infarct and healthy tissue respond differently to tissue processing.

A key component of any study aimed at investigation of infarct healing is characterization of the processes involved, including inflammatory cell recruitment and neovascularization, both commonly detected in tissue sections by immunohistochemistry (9, 16). Experiments conducted here demonstrate that tissue that has undergone processing for OPT retains its integrity and immunoreactivity, permitting detection of CD31 and Mac-2 for identification of endothelial cells and macrophages respectively. OPT can facilitate this kind of investigation by identifying the most appropriate area of the infarct for tissue collection and analysis. In developmental studies specific antibodies have been used to detect proteins of interest and their distribution within the reconstructed embryo (22). Within the heart antibody penetration is likely to be more limited, but the potential exists for whole tissue staining of the vascular endothelium that can be accessed during myocardial perfusion and this merits further investigation.

As with other methods OPT has its limitations. Ironically, while it is the strong autofluorescence of heart tissue that enables OPT imaging to acquire such good quality images this also limits detection of e.g., green or red fluorescent protein, or of exogenous antibody-conjugated fluorescent probes. There are various protocols to quench autofluorescence (1, 2), and it may be possible to apply these to improve detection of specific proteins. Tissue must also be optically cleared prior to scanning, and although the lipophilic agent (benzyl benzoate/benzyl alcohol) used for this purpose is reported to cause minimal deformation of tissue morphology (4), it can remove lipid pools from tissue. While this is an issue shared with standard histology, where tissue preparation can also remove lipid, it may limit the use of OPT for some applications. The image quality achieved is superior to that of other 3D imaging techniques, and even comparable to histological sections. However, it could also be improved further, perhaps by refinement of the back-projection algorithms used to reconstruct OPT data sets (28, 29).

In conclusion, this study demonstrates that OPT imaging is a nondestructive, accurate approach for routine assessment of murine myocardial injury in 2D and 3D. The data generated correlate well with conventional 2D histological approaches but permit calculation of infarct volume that correlates with volumes generated by MRI. Application of OPT may help to reduce the number of animals required to detect differences in infarct size in response to therapeutic intervention. While there are some current limitations, OPT has the potential to be applied beyond detection of tissue injury, for example in 3D assessment of anatomical features such as cardiac valve stenosis. Developments in this area and in detection of fluorescent proteins within the myocardium hold significant promise.

## GRANTS

This work was supported by the British Heart Foundation Centre of Research Excellence award and by grants from China Scholarship Council/University of Edinburgh Scholarship to X. Zhao, British Heart Foundation (PG/12/74/29745 to M. A. Jansen), and the Wellcome Trust (WT 091720MA to G. A. Gray).

## DISCLOSURES

No conflicts of interest, financial or otherwise, are declared by the author(s).

## AUTHOR CONTRIBUTIONS

Author contributions: X.Z. and G.A.G. conception and design of research; X.Z., J.W., K.M., and M.A.J. performed experiments; X.Z. analyzed data; X.Z., J.W., C.D.G., and G.A.G. interpreted results of experiments; X.Z. and H.M. prepared figures; X.Z. and G.A.G. drafted manuscript; X.Z., J.W., C.D.G., A.G.R., H.M., M.A.J., and G.A.G. edited and revised manuscript; X.Z. and G.A.G. approved final version of manuscript.

## Supplementary Material

Video S1

Image S2

## Footnotes

1 The online version of this article contains supplemental material.
