# Supplementary figures and images for: Optical projection tomography permits efficient assessment of infarct volume in the murine heart postmyocardial infarction
Source: Am J Physiol Heart Circ Physiol. 2015 Jun 12;309(4):H702–10. doi: 10.1152/ajpheart.00233.2015 (PMC4537945; doi:10.1152/ajpheart.00233.2015)

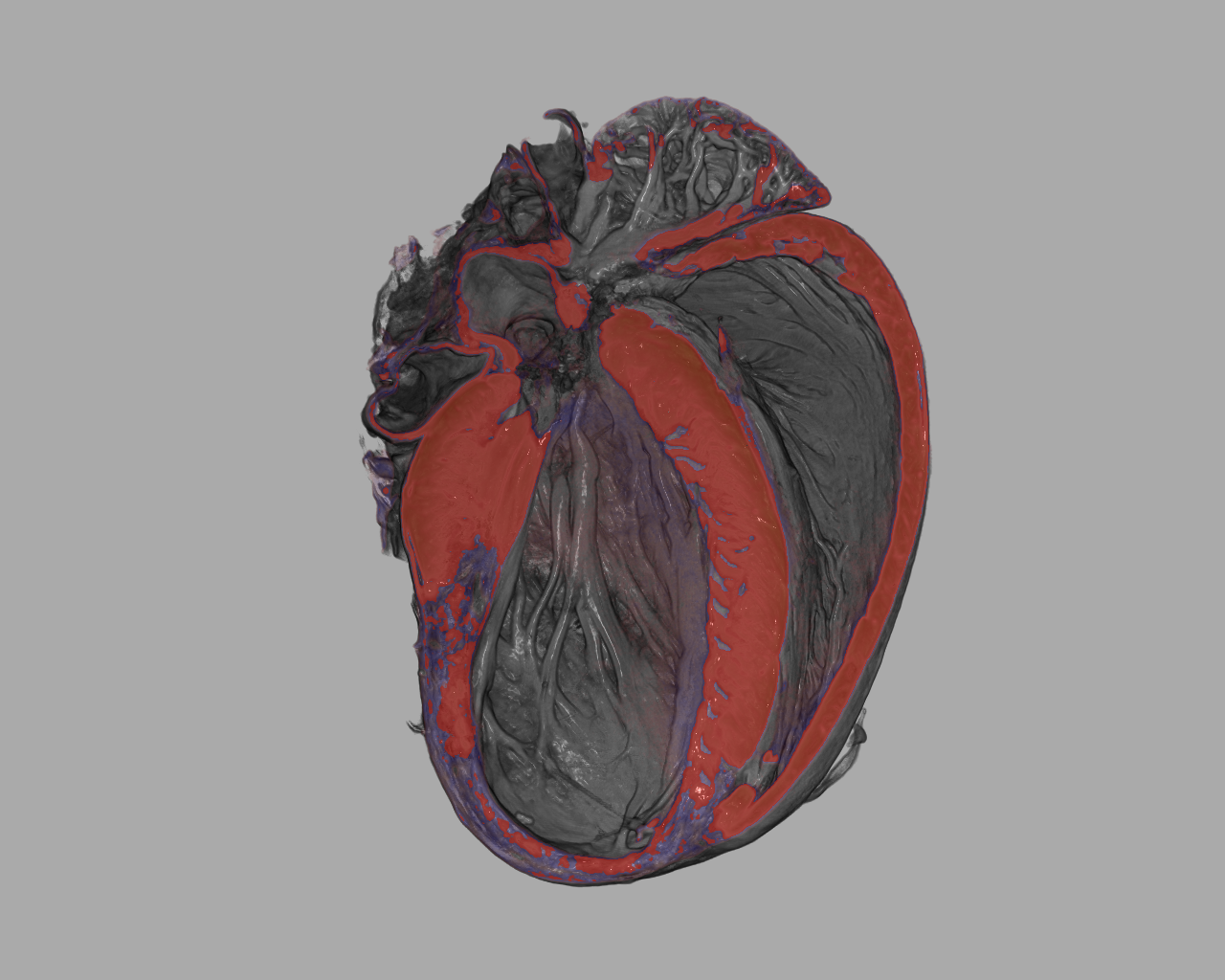

Supplement: Image S2 [file Image_S2.png]
